# Archaeal Lipids: Extraction, Separation, and Identification via Natural Product Chemistry Perspective

**DOI:** 10.3390/ijms26073167

**Published:** 2025-03-29

**Authors:** Tuo Li, Youyi Luo, Changhong Liu, Xuan Lu, Baomin Feng

**Affiliations:** College of Life and Health, Dalian University, Dalian 116622, China; 081114lyy@sina.com (Y.L.); liuchanghongnb@163.com (C.L.); luxuan_232@163.com (X.L.); fbmdlu@163.com (B.F.)

**Keywords:** archaea, lipids, extraction, separation, identification, natural product chemistry

## Abstract

Archaeal lipids, defining a primordial life domain alongside Bacteria and Eukarya, are distinguished by their unique glycerol-1-phosphate backbone and ether-linked isoprenoid chains. Serving as critical geochemical biomarkers, archaeal lipids like glycerol dialkyl glycerol tetraethers (GDGTs) underpin paleoclimate proxies, while their phylum-specific distributions illuminate phylogenetic divergence. Despite the maturity of Mass Spectrometry-based quantitative biomarkers—predominantly those with established structures—becoming well-established in geochemical research, systematic investigation of archaeal lipids as natural products has notably lagged. This deficit manifests across three key dimensions: (1) Extraction methodology lacks universal protocols adapted to diverse archaeal taxa and sample matrices. While comparative studies exist, theoretical frameworks guiding method selection remain underexplored. (2) Purification challenges persist due to the unique structures and complex isomerization profiles of archaeal lipids, hindering standardized separation protocols. (3) Most critically, structural characterization predominantly depends on decades-old foundational studies. However, the existing reviews prioritize chemical structural, biosynthetic, and applied aspects of archaeal lipids over analytical workflows. This review addresses this gap by adopting a natural product chemistry perspective, integrating three key aspects: (1) the clarification of applicable objects, scopes, and methodological mechanisms of various extraction technologies for archaeal lipids, encompassing both cultured and environmental samples; (2) the elucidation of separation principles underlying polar-gradient lipid fractionation processes, leveraging advanced chromatographic technologies; (3) the detailed exploration of applications for NMR in resolving complex lipid structures, with specialized emphasis on determining the stereochemical configuration. By synthesizing six decades of methodological evolution, we establish a comprehensive analytical framework, from lipids extraction to structural identification. This integrated approach constructs a systematic methodological paradigm for archaeal lipid analysis, bridging theoretical principles with practical implementation.

## 1. Introduction

**Figure 1:**
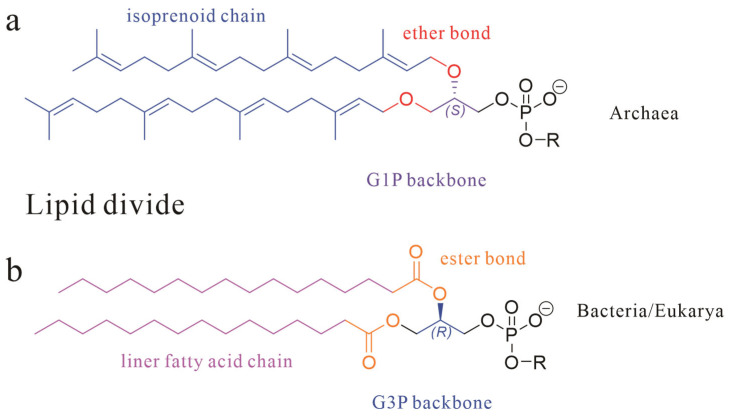
The basic skeletal structure of phospholipids in Archaea (**a**) and Bacteria and Eukarya (**b**); three core dimensions are marked with different colors.

Archaea, one of the three fundamental domains of life (alongside Bacteria and Eukarya), exhibit cellular membranes that, while architecturally homologous to bacterial membranes in their amphiphilic lipid–protein framework, display profound molecular-level divergence [1,2]. Specifically, archaeal lipid characteristics can be distinguished from those of Bacteria/Eukarya through three core dimensions as shown in Figure 1: stereochemical configuration (sn-1 vs. sn-3 glycerol backbone), chemical bond type (ether vs. ester linkages), and hydrophobic unit properties (isoprenoid chains with isomerization vs. straight-chain fatty acids), collectively termed the “lipid divide” [3,4,5].

These molecular distinctions manifest structural consequences: for instance, thermophilic Crenarchaeota develop monolayer membranes stabilized by the covalent tethering of tetraether alkyl chains, a structural adaptation enabling survival under extreme conditions [6,7,8]. As chemical markers of microbial ecology, archaeal polar lipids hold dual scientific significance. Their geochemical utility is epitomized by lipids like glycerol dialkyl glycerol tetraethers (GDGTs), whose cyclization patterns underpin the TEX_86_ paleothermometer for reconstructing paleoclimatology [9,10,11,12].

Simultaneously, lipid profiles reflect phylogenetic divergence [13,14,15]. For instance, GDGTs are found in Euryarchaeota but are systematically absent in halophilic archaea, highlighting lipid-based taxonomic utility despite interspecies variability [16,17,18,19]. This interplay between biological traits and geochemical signatures positions archaeal lipid research as a critical nexus between geology and life science [20,21].

**Figure 2:**
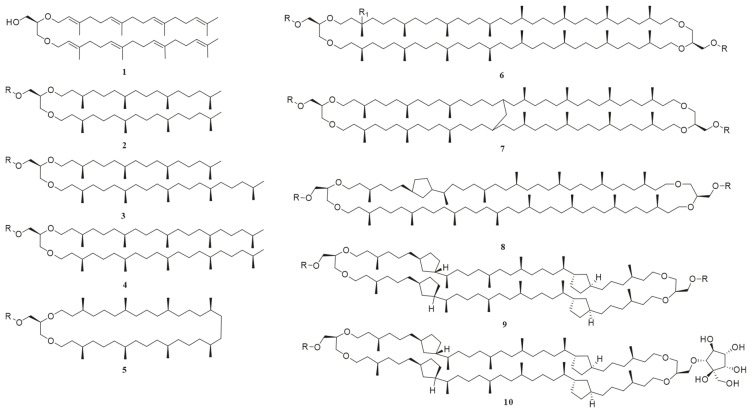
The chemical structures of representative archaeal lipids, where the *R* group in all compounds is either a polar head group or H; R = H, compounds **1**–**5** are diether lipids: **1**: *sn*-2,3-di-*O*-geranylgeranylglyceryl alcohol (DGGGOH); **2**: 2,3-di-*O*-phytanyl-*sn*-glycerol; **3**: 2-*O*-sesterpanyl-3-*O*-phytanyl-*sn*-glycerol; **4**: 2,3-di-*O*-sesterpanyl-*sn*-glycerol; and **5**: macrocyclic diether. Compounds **6**–**9** are GDGTs: R_1_ = H; **6**: GDGT-0; **7**: GMGT-0; **8**: GDGT-1; **10**: backbone of glycerol dialkyl nonitol tetraether (GDNT); and **9**: GDGT-4; R_1_ = OH; **6**: hydroxy-GDGT-0.

Foundational discoveries in archaeal lipid chemistry predated the domain’s taxonomic recognition. In 1962, 15 years before Carl Woese proposed archaea as a separate domain [22], Kates and colleagues isolated the first diether phospholipid from *Halobacterium cutirubrum* [23]. Over subsequent decades, over 100 novel ether-linked polar lipids (phospholipids, glycolipids, and phosphoglycolipids) were characterized (Figure 2) [4,7,16,17,18,24]. The earliest documented record of the chemical structures of archaeal lipids dates back to 1988 [24], while the second review article addressing this topic was published in 2013 [17]. Notably, a comprehensive review by Zoe E. Wilson and Margaret A. Brimble in the *Natural Product Reports* (2009) specifically focused on natural products derived from extremophiles, including lipids from thermophilic archaea [25]. In their 2021 update, which compiled natural products derived from extremophiles over the 2008–2019 research period, the latest publication unfortunately did not provide comprehensive coverage of archaeal lipids [26]. Yet, existing reviews largely focus on chemical structures, biosynthetic pathways, or biotechnological applications [4,18,27,28] and their discussions predominantly focus on established mature technical systems with standardized protocols, particularly mass spectrometry-based analytical platforms. Yet, critical methodological advancements extensively documented in the literature—including multi-strategy lipids extraction methods (such as combined mechanical disruption and solvent extraction techniques) and applications of nuclear magnetic resonance (NMR) spectroscopy in resolving complex lipid stereochemistry—have not yet received systematic examination. This leaves a critical gap: the absence of a unified framework integrating natural product chemistry workflows, spanning optimized sample preparation and extraction protocols, through refined separation techniques, to structural elucidation.

This review pioneers a natural product chemistry-centric perspective on archaeal lipids. It addresses the following: (1) extraction strategies contrasting pure cultures with environmental samples, (2) advances in chromatographic separation, and (3) NMR-driven structural elucidation. By synthesizing decade-spanning methodologies, we aim to propose a standardized framework from sample collection to structural elucidation of archaeal lipids, while illuminating untapped potentials in geochemical tracing and synthetic biology.

## 2. Extraction Methodologies and Applications of Archaeal Lipids

In this section, we present a detailed overview by compiling and organizing the extraction methods from the ‘Methods’ Section of various literature sources. During the organization process, we noticed that authors frequently neglect to justify their reason for choosing one extraction protocol over another. Thus, given the differences in extraction strategies and technical challenges across sample matrices, subsequent sections will elaborate on and discuss their respective processing protocols.

### 2.1. Sample Pretreatment

Sample pretreatment serves as the foundation of lipid analysis, playing a pivotal role in lipid extraction by enabling efficient lipid recovery [29,30,31,32]. This review focuses on archaeal lipid extraction through two distinct approaches based on sample origin: (1) targeted extraction from pure archaeal cells and (2) comprehensive extraction from environmental matrices (including sediments, seawater, and biological substrates).

#### 2.1.1. Processing of Pure Culture Cells

The principal technical challenge in archaeal lipid extraction from pure cultures resides in achieving efficient cellular membrane disruption. The established methodologies, adapted from microbial lipid extraction paradigms, are classified into three categories: mechanical lysis (ultrasonication, high-pressure homogenization, and freeze–thaw cycles), chemical dissolution (surfactant-based permeabilization), and enzymatic degradation [33,34]. These techniques have been extensively documented in numerous lipid studies and, thus, will not be reiterated here.

However, there are still a few recommendations worth mentioning in this section. Ultrasonic disruption represents the preferred methodology for small-scale archaeal cell lysis [35], whereas high-pressure homogenization demonstrates superior throughput efficiency in high-density archaeal cultures. Methodological selection should be optimized through the empirical validation of laboratory instrumentation capabilities and biomass parameters. Notably, archaeal lipid composition exhibits marked sensitivity to cultivation parameters, particularly in extremophilic taxa. For instance, research by Yasuhiko Matsuno et al. demonstrates that the membrane lipid composition of *Thermococcus kodakaraensis* undergoes dynamic regulation, with the ratio of diphytanylglycerol diether to dibiphytanyldiglycerol tetraether being modulated in response to both growth phase and cultivation temperature [36]. Another study showed that three critical parameters—growth phase, medium pH, and cultivation temperature—demonstrate significant correlations with GDGT compositional dynamics in the *Picrophilus torridus* strain [37]. These observations underscore the imperative for rigorous parameter standardization in experimental cultivation protocols.

#### 2.1.2. Processing of Environmental Samples

Post collection, environmental samples are typically subjected to cryopreserve in liquid nitrogen and maintained at ultra-low temperature conditions (−80 °C or below) to preserve their molecular integrity. Pretreatment strategies must be tailored to research objectives: whether targeting viable archaeal populations or sedimentary/fossilized core lipids. Aqueous samples undergo sequential filtration through graded membranes (60 μm nylon → 0.7 μm GFF (glass fiber filters) → 0.2 μm Sterivex filter) [38]. Recent investigations have identified the 0.2–0.7 μm fraction as particularly enriched with archaeal-derived GDGTs [39,40,41].

For solid matrices (sediments and soils), lyophilization is commonly performed first, followed by mechanical homogenization. However, reports indicate that freeze-drying reduces the recovery of isoprenoid GDGTs (I-GDGTs) in both Durapore^®^ and GFF compared to air-drying, a phenomenon related to the oxidative loss mechanism of phospholipid substances [42]. For viable archaeal membrane lipid preservation, cryogenic grinding (under liquid nitrogen) is recommended.

Additionally, several publications emphasize the need for the rigorous decontamination of experimental equipment. Glassware should be calcined in a muffle furnace at 400–500 °C for 6 h, while plastic items should be cleaned with dichloromethane (DCM) using ultrasonication [39,43]. For specific samples, saponification pretreatment (e.g., 0.3 M potassium methoxide, 70 °C for 1 h) may also be required to dissociate lipoprotein complexes [34,42,44].

### 2.2. Liquid–Liquid Extraction

**Figure 3:**
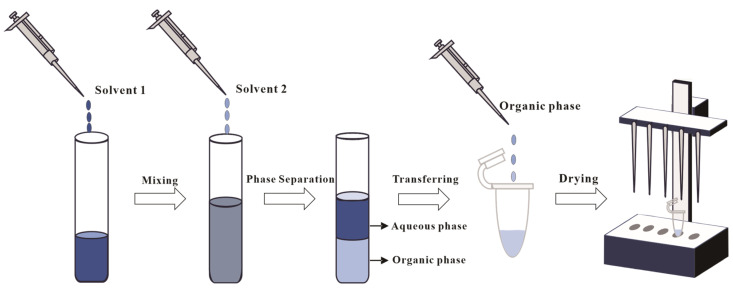
Illustration of the Bligh and Dyer method. Solvent 1 is a mixture of chloroform (or DCM) and methanol (1:2, *v*/*v*) or a triphasic solvent system with methanol, chloroform (or DCM), and water (e.g., 2:1:0.8, *v*/*v*/*v*), and solvent 2 is a chloroform (or DCM) and water mixture (1:1, *v*/*v*).

The modified Bligh–Dyer (B&D) method remains the predominant liquid–liquid extraction (LLE) for archaeal lipid extraction in both pure cultures and environmental samples [45,46,47,48]. Two primary modified B&D methods have emerged: one predominantly utilized for the extraction of lipids from halophilic archaea [49], and the other constituting an optimized approach tailored for other archaeal groups (e.g., thermoacidophilic species) [42]. The fundamental difference between these two methods resides in the initial solvent composition: the halophilic protocol typically employs a binary organic solvent mixture of chloroform (or DCM) and methanol (1:2, *v*/*v*), whereas the alternative method incorporates a triphasic solvent system comprising methanol, chloroform (or DCM), and water (e.g., 2:1:0.8, *v*/*v*/*v*). In the subsequent extraction, both modified methods involve a secondary addition of a chloroform (or DCM) and water mixture (1:1, *v*/*v*). As illustrated in Figure 3, phase separation through gravitational settling or centrifugation yields lipid-enriched chloroform (or DCM) fractions.

Low-toxicity alternatives such as dichloromethane/methanol have been developed to replace traditional chloroform/methanol systems, often combined with phosphate-buffered aqueous phases [42,43]. Despite improvements, limitations persist in polar lipid recovery (particularly intact polar lipids [IPLs]) and GDGT losses. To address this, the acidification of the aqueous phase is conducted to improve archaeal lipid yields. However, strong acids like HCl can cause the hydrolysis of intact membrane lipids (polar headgroup degradation), leading to the subsequent adoption of trichloroacetic acid (TCA) [42,43,50]. Moreover, achieving precise recovery of the chloroform organic layer (lower layer) without cross-contamination proves technically demanding.

Another important membrane lipid extraction method is the Matyash method, which uses methyl tert-butyl ether (MTBE) as the extraction solvent. Its advantage lies in the enrichment of lipids in the upper organic phase, and experimental data indicate that MTBE has a 15–20% higher recovery rate for polar lipids than the B&D method [41,51]. However, the high volatility of MTBE poses challenges for experimental reproducibility.

Recently, Evans et al. demonstrated that cetyltrimethylammonium bromide (CTAB)-assisted lysis with thermal cycling enhances cultured archaeal lipid yields 2.3-fold, though environmental responses varied (marine particulates: 1.7-fold vs. sediments: no significant difference) [34]. Additionally, an earlier report indicated that archaeal cells subjected to high-pressure homogenization exhibit lipid extraction rates comparable to those of untreated cells using acidified extraction methods [50]. These findings suggest that cell lysis is an effective method for extracting intact lipids from live archaeal cells.

### 2.3. Soxhlet Extraction

Soxhlet extraction, originally devised by Franz von Soxhlet in 1879, was initially employed for determining the lipid content of dairy products; it operates through an evaporation–condensation–siphoning mechanism [33,52]. In this system, homogenized dried samples in porous thimbles undergo continuous extraction via cyclic organic solvent permeation. By recycling limited solvent volumes, this method enhances extraction efficiency particularly suited for thermally stable compounds [33]. The Soxhlet extraction protocol for archaeal lipids typically employs mixed solvent systems (DCM/MeOH = 9:1 or 2:1, *v*/*v*) under thermal conditions (over 60 °C) for 24–72 h [42,53,54,55,56]. Some studies involve either solvent evaporation or phase separation via 5% KCl or NaCl solution partitioning, followed by the DCM re-extraction of aqueous phases and subsequent organic phase concentration [53,54]. In the study by Sabine K. Lengger et al., it was found that Soxhlet extraction and B&D extraction yielded comparable quantities of lipids. However, Soxhlet extraction demonstrated significant bias toward certain intact polar lipid GDGTs (IPL GDGTs), particularly those containing hexose-phosphohexose headgroups (HPH GDGTs). This bias may be attributed to the thermal sensitivity of these compounds, which could lead to instability during the extraction process due to temperature fluctuations [55].

In recent years, instruments enabling automated or semi-automated Soxhlet extraction have been developed and introduced, which can enhance extraction kinetics and significantly reduce extraction time and solvent consumption [31]. Notably, microalgal lipid research provides transferable optimization paradigms [57]. For example, Ramluckan et al. studied thirteen solvents with different polarities, and achieved about 11.76% yield enhancement in *Chlorella vulgaris* lipid recovery through chloroform/ethanol (1:1) polarity optimization [58], while Aravind et al. reported 83% extraction yield in 50 g of *Spirulina platensis* through sample pretreatment strategies [59]. Nevertheless, critical knowledge gaps persist regarding archaeal-specific adaptations, particularly in low-toxicity solvent compatibility (n-hexane/isopropanol systems) and polar headgroup stabilization-key priorities for future methodological development.

### 2.4. Accelerated Solvent Extraction

Accelerated solvent extraction (ASE) represents a novel extraction technique that enhances compound solubilization via dual mechanisms: (1) temperature-induced solvent viscosity reduction coupled with elevation in solute diffusivity; (2) pressure-mediated liquid-state solvent maintenance (supra-boiling point conditions) achieving matrix permeability enhancement [60]. Standard ASE instrumentation integrates a pressurized extraction cell (1–100 mL), precision thermal regulators, solvent infusion pumps, and inert gas purgers for automated operation [31,61]. In microalgal lipid extraction applications, ASE has demonstrated remarkable efficacy. Tang et al. reported a 6.9% yield enhancement in *Chlorella vulgaris* lipids using four static cycles at 100 °C (total duration < 30 min) [62]. The methanol/Dimethyl Sulfoxide (DMSO)-hexane/ether composite system developed by the Chen team achieved lipid extraction from multiple algae species in just 3 min at 125 °C, reducing solvent consumption by 58% compared to the B&D method [63]. Environmental analyses have greatly benefited from the implementation of ASE, as demonstrated by Jeannotte et al.’s study where four mixed organic solvents were used to assess the extraction efficiency of soil lipids. Their findings revealed that the combination of buffer or chloroform–methanol mixtures resulted in the highest extraction yields of soil lipids using the ASE system [64]. In the study by K. Quénéa et al., the efficacy of varying solvents and temperatures for extracting soil lipids from two distinct sources was systematically compared using ASE [65].

In recent years, ASE technology has also shown unique advantages in the extraction of archaeal lipids, particularly GDGTs from environmental matrices. Huguet et al. successfully extracted GDGTs from settled particulate matter and sediment cores using a DCM/MeOH (9:1) solvent system at 100 °C with 7.6–10.6 megapascals (MPa) [42]. Kim’s team validated method robustness through ASE pretreatment of 287 globally distributed marine surface sediments (DCM/MeOH = 9:1) [10]. Auderset et al. innovated a polarity-gradient approach employing sequential hexane-DCM-DCM/MeOH (1:1) elution, enabling concurrent extraction of n-alkanes and alkenones from marine/lacustrine sediments with enhanced precision for U^K^_37_ indices at low concentrations [66]. The recently developed Large-Volume Injection–Liquid Chromatography-Atmospheric Pressure Chemical Ionization—Mass Spectrometry (ASE-LVI-LC-MS) protocol utilizes DCM/MeOH (3:1) at 80 °C (3 cycles), reducing sample requirements to 0.02–0.10 g while maintaining exceptional precision (Relative Standard Deviation [RSD] ≤ 10.2%) and linearity (*R*^2^ ≥ 0.991) for GDGTs analysis in marine particulates [67].

Despite these advantages, ASE implementation presents three primary limitations: (1) thermally sensitive components can exhibit degradation at temperatures > 100 °C; (2) a degassing system is necessary to prevent lipid oxidation; (3) the initial investment cost for the equipment is relatively high. For thermally unstable samples, a staged temperature strategy is recommended: initially using low temperatures to solubilize lipids, followed by higher temperatures to extract membrane-bound lipids, thereby balancing extraction efficiency and compound integrity [31,68].

### 2.5. Saponification Extraction

Saponification is an alkaline hydrolysis process where fats or oils undergo reaction with hydroxides (e.g., KOH or NaOH) to yield, for example, glycerol and soap derivatives [69]. While this extraction technique is routinely employed for fatty acid isolation from environmental or microbial samples [57,69], its application in archaeal lipid extraction is limited due to the characteristic ether linkage between side chains and glycerol moieties. However, the polar head groups in archaeal lipids are connected to the glycerol backbone through phosphate ester bonds, which are susceptible to alkaline hydrolysis. In geochemical investigations of environmental or fossilized samples, archaeal lipids often retain only core structures due to natural hydrolysis of polar head groups, further negating the need for saponification. Despite the limitations, saponification plays a critical role in archaeal lipid analysis, particularly in resolving side-chain architectures. Hydrolysis of phosphate head groups generates alcohol derivatives, which can be detected via gas chromatography (GC) or GC-mass spectrometry (GC-MS) after derivatization [70].

**Figure 4:**
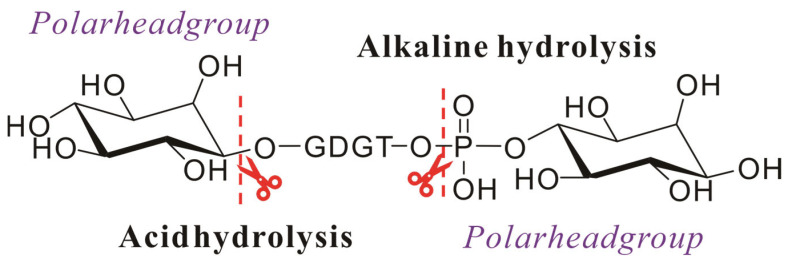
Schematic diagram of the hydrolysis of the intact polar archaeal lipids: ester bonds are hydrolyzed under alkaline conditions, and ether bonds are hydrolyzed under acidic conditions.

Following Huguet et al.’s validated methodology, samples were initially refluxed with 1 M KOH (1 h) for alkaline hydrolysis (to cleave phosphate esters), acidified to pH 3 with HCl, and extracted with DCM. Subsequent acid hydrolysis (1 M HCl, 5 h, disruption of ether linkages) of the aqueous phase yielded GDGTs through head group elimination (Figure 4) [42]. In addition, the initial extraction step achieved higher core lipid yields, likely due to saponification-induced dissociation of lipid–protein complexes, enhancing extraction efficiency. However, studies utilizing alternative extraction protocols demonstrated that saponification-based methods exhibited significantly reduced efficacy compared to conventional techniques for isolating archaeal membrane lipids from environmental matrices. Thus, compared to extracting lipids from environmental samples, saponification-based extraction methods are more suitable for lipids from pure cultures of archaea.

### 2.6. Ultrasound-Assisted Extraction

Ultrasonic-assisted extraction (UAE) functions via cavitation dynamics and mechanical oscillation, employing probe or bath configurations to induce transient microbubble formation [71]. Subsequent bubbles near solid surfaces at elevated amplitudes disrupt cellular structures, accelerating the release of intracellular compounds into solvents. Chemat et al. systematically characterized synergistic mechanisms including fragmentation, erosion, sonocapillary effects, and shear force generation [72]. Compared to conventional methods, ultrasound-enhanced extraction demonstrates superior efficiency through intensified mass transfer, with broad applications spanning food processing, nutraceuticals, cosmetics, pharmaceuticals, and bioenergy production.

In the context of archaeal lipids extraction, UAE is often combined with the B&D method for extracting lipids from environmental samples. The modified protocol involves sonicating the primary extraction solvent (typically chloroform or DCM–methanol mixtures) for 5–15 min in three cycles prior to phase separation steps [38,73,74,75]. Wang et al.’s comparative study revealed distinct GDGT recovery profiles: ultrasonic solvent extraction preferentially liberated core GDGTs from soil and lacustrine sediments, whereas conventional B&D methods yielded higher intact polar GDGTs [76]. Meanwhile, the application of UAE for archaeal lipids presents unresolved methodological constraints, notably the unoptimized relationships between extraction efficiency and critical operational parameters (e.g., temperature modulation and frequency-dependent cavitation thresholds). The systematic characterization of these variables is essential to maximize lipid recovery and reproducibility.

It is noteworthy that UAE and ultrasonic cell disruption are two distinct techniques. For instance, in the extraction of membrane lipids from cells, the ultrasonic equipment used in UAE, such as ultrasonic baths, generally does not have sufficient power to disrupt the structure of archaeal cells. In contrast, ultrasonic cell disruption employs power-adjustable ultrasonic probes that can generate intensities sufficient to disrupt cell structures [35,74]. Thus, theoretically, the integration of B&D solvents with ultrasonic cell disruption could synergistically enhance efficiency as the combined effects of organic solvent polarity and cavitation-induced cell lysis may facilitate superior lipid solubilization and release. Disruptive ultrasonication falls within the pretreatment section and will not be discussed extensively here.

### 2.7. Solid-Phase Extraction

Solid-phase extraction (SPE) can be considered a separation technique, and although it is occasionally utilized for the extraction of archaeal lipids, a detailed discussion on this topic will be provided in the subsequent Section.

## 3. Separation and Purification Strategies for Archaeal Lipids

Following archaeal lipid extraction, subsequent separation protocols demand the strategic adaptation of the established chromatographic techniques, with key methodologies including column chromatography (CC), high-performance liquid chromatography (HPLC), thin-layer chromatography (TLC), and SPE, among others, with the core differences lying primarily in the selection and optimization strategies of the elution systems.

For environmental matrices that often harbor archaeal–bacterial consortia, integration with structural characterization via HPLC coupled with MS typically substitutes discrete off-line purification steps, though necessitating rigorous contaminant verification against co-extracted bacterial lipids. Conversely, the analysis of pure archaeal cultures needs complete compound separation to ensure target lipid purity for subsequent structural elucidation and functional characterization, with methodological selection contingent upon experimental objectives: streamlined analytical workflows versus exhaustive preparative isolation.

### 3.1. Column Chromatography

As a fundamental separation technique, CC achieves the differential retention of compounds through the synergistic effect of the stationary phase (e.g., silica gel and alumina) and the mobile phase (eluent). Silica gel is the most widely used adsorbent accounting for 90% of plant chemical component separations, whereas alumina’s application has declined due to side reaction risks (dehydration, decomposition, and isomerization) despite its superior polarity [77].

**Figure 5:**
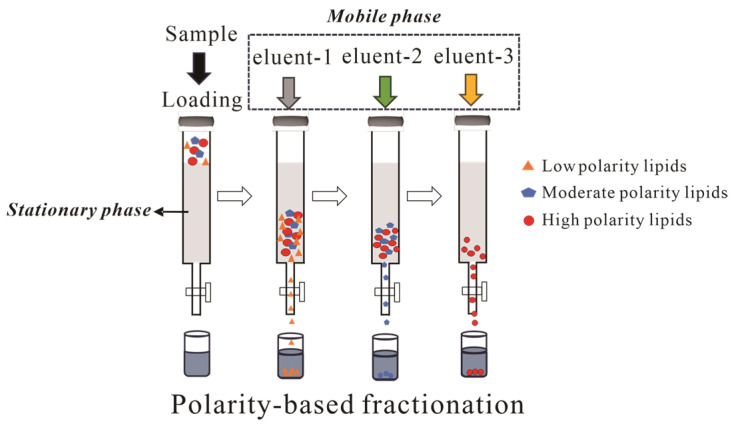
Schematic diagram of archaeal lipids using column chromatography.

Archaeal lipid fractionation typically employs sequential elution with polarity gradients as shown in Figure 5. A representative protocol by Chun Zhu et al. utilizes silica gel with hexane → hexane: DCM (1:4 *v*/*v*) → DCM → methanol, yielding GDGTs in tertiary and quaternary fractions [78]. Alternative protocols utilizing hexane–DCM (9:1 *v*/*v*) and DCM–methanol (1:1 *v*/*v*) achieve distinct polarity-based fractionation, while alumina columns similarly concentrate GDGTs into polar fractions [75]. Pitcher et al. identified limitations in conventional DCM/acetone/methanol gradients for resolving core GDGTs from glycosylated derivatives. Their optimized hexane–ethyl acetate (3:1 *v*/*v*) → ethyl acetate → methanol protocol achieved both high recovery and effective core GDGTs/IPL separation in geothermal soils and marine particulates. Subsequent analyses revealed significant qualitative and quantitative differences between core GDGTs and IPL-GDGTs, validating their utility for fossil biomarker studies [79].

The separation of archaeal lipids requires a systematic evaluation of multiple interdependent parameters, including but not limited to ① stationary phase selection (silica’s polar group stability vs. alumina’s reactivity); ② mobile phase optimization (balancing separation efficiency and compound integrity); ③ multi-step gradients (typically 3 to 4 solvents for polarity-based sequential elution).

### 3.2. High-Performance Liquid Chromatography

The chromatographic resolution of high-performance liquid chromatography (HPLC) critically depends on the synergistic optimization of stationary phase selection (e.g., HPLC column) and mobile phase composition. Normal-phase (NP) and reverse-phase (RP) configurations, when coupled with tailored mobile phases, provide robust platforms for complex lipid resolution through distinct retention mechanisms. NP–HPLC operates on a separation mechanism analogous to conventional CC using silica gel or alumina, as demonstrated in Figure 5. Conversely, RP–HPLC exhibits an inverse separation paradigm, utilizing hydrophobic stationary phases (commonly C18 or C8 alkyl chains bonded to silica) with polar mobile phase systems, resulting in reversed elution order where more nonpolar lipid species are eluted first. Recently, review articles have elaborated HPLC parameter-selection strategies for archaeal lipid separation [4,80], rendering redundant discussions of conventional column/mobile phase combinations in this review.

In addition to the established HPLC approaches for archaeal lipid separation, the development of chiral separation protocols is now driven by the need for rapid stereochemical characterization of the lipids. The stereochemical dichotomy between archaeal and bacterial/eukaryotic membrane lipids—manifested through *S*-configuration glycerol-1-phosphate (G1P) versus *R*-configuration glycerol-3-phosphate (G3P) backbones—presents critical challenges in chiral resolution. Palyzová et al. demonstrated bacterial membrane heterochirality through integrated GC–MS and LC–MS analyses, detecting 20–30% *S*-configuration isomers in phosphatidylglycerol backbones of *Bacillus amyloliquefaciens*, *B. subtilis*, *Clavibacter michiganensis*, and *Geobacillus stearothermophilus* [81]. Remarkably, this ratio aligns with the tolerance threshold observed in engineered *Escherichia coli* incorporating archaeal lipids, suggesting the potential biological viability of heterochiral membranes. Their chiral HPLC protocol, though requiring extended run times (~150 min), provided critical evidence for bacterial heterochiral membrane hypotheses. The subsequent application of chiral separation technology revealed that *sn*-2,3-di-*O*-geranylgeranylglyceryl alcohol (DGGGOH) intermediates in archaeal lipid biosynthesis within *Saccharomyces cerevisiae* and *E. coli*, unveiling the enzymatic capacity for archaeal-configuration synthesis and offering novel evolutionary insights [82].

### 3.3. Thin-Layer Chromatography

TLC, an early liquid chromatography variant, remains pertinent for lipid analysis through differential adsorption affinities on silica gel stationary phases [83]. Two-dimensional TLC (2D TLC) enhances resolution through orthogonal separation mechanisms, capitalizing on the technique’s operational advantages: procedural simplicity, cost-effectiveness, and rapid separation—particularly advantageous for post-cultivation archaeal lipid analysis [84]. The TLC workflow involves applying samples about 2 cm from the TLC plate edge via microsyringes, followed by chamber development with optimized solvent systems. Visualized via UV-active dyes or lipid-specific sprays, component identification relies on *Rf* value comparison against standards [85]. Target bands are then scraped and extracted, for example, using cold methanol precipitation, for further downstream analyses. Most polar lipids separated by TLC can be easily identified using characteristic color reactions, with a plethora of spray reagents providing specific color responses that will be discussed in the subsequent section.

In 2D TLC analysis of archaeal polar lipids, the developing solvent systems typically exhibit distinct polarity characteristics, with the first-phase developing solvent generally demonstrating low polarity and the second phase being relatively higher. The selection of currently employed developing solvent types also draws from analytical experience with other microbial lipids. Specifically, the first-phase developing solvent consists of chloroform, methanol, and water in a volume ratio of 65:25:4. The second phase may utilize a mixture of chloroform, acetic acid, methanol, and water in a volume ratio of 80:18:12:5, or a slightly modified ratio of 80:15:12:4. Additionally, another commonly used developing solvent system employs a first phase composed of chloroform, methanol, acetone, and acetic acid (90:10:6:1 by volume), while the second phase adopts a mixture of chloroform, methanol, acetic acid, and water (100:20:12:5 by volume) [86].

Following separation, individual TLC spots are typically scraped off for analysis, enabling the rapid isolation of pure membrane lipids and facilitating structural identification. For instance, researchers have successfully utilized prep-TLC in combination with FAB–MS to isolate and identify the core lipids present in 17 different strains of *Thermococcales* [87]. TLC has proven effective in separating simple lipid classes, such as monoacylglycerols. For example, genus-level taxonomic differentiation in archaea exploits TLC-derived polar lipid migration patterns, with 1D/2D TLC being recommended for novel halophilic archaeon characterization [88]. For complex lipid samples, further chromatographic separation can be achieved using 2D TLC technology. Additionally, the successful application of TLC in conjunction with techniques like Matrix-Assisted Laser Desorption/Ionization Mass Spectrometry (MALDI-MS) has yielded highly promising results [89,90,91,92].

Focusing specifically on archaeal lipid research, TLC serves dual functions: (1) taxonomic differentiation based on genus-specific polar lipid migration patterns, and (2) rapid fractionation of target lipid classes (e.g., GDGTs) from complex total lipid extracts through optimized solvent systems.

### 3.4. Solid-Phase Extraction/Separation

SPE is a widely used technique for lipidomics, leveraging liquid–solid chromatography principles to achieve the selective isolation of target analytes from complex matrices [93,94]. This technology operates through differential affinity interactions between lipid species and stationary phases (adsorbents), fulfilling dual critical roles in lipid research: (1) selective separation and purification of specific lipids and (2) preconcentration of trace lipid biomarkers. Operational workflows comprise four optimized stages: sorbent activation, sample loading, impurity washing, and target elution, implementable through reverse-phase (C18/C8/C2), normal-phase (silica/amino/cyano), or ion-exchange (strong anion/cation) mechanisms [94].

SPE’s efficiency arises from a dual mechanism: initial coarse separation dominated by size exclusion effects, followed by the fine-tuning of target lipid retention through hydrophilic–hydrophobic interactions [94]. Notably, while SPE is recognized for its high degree of automation and rapid sample preparation, subtle deviations in operational parameters, such as elution flow rate and solvent polarity, can lead to significant inter-batch variability. Compared to the oxidation degradation risks associated with TLC and the inefficiency and time-consuming nature of traditional CC, SPE offers clear advantages in terms of standardization and reproducibility.

Rong Zhu et al. reported that the recovery of phospholipid using HybridSPE^®^ is superior to that achieved through silica CC, with concurrent 1.2–2.7-fold LC–Electrospray Ionization (ESI)–MS signal enhancement in marine sediments [95]. Xiaolei Liu et al. effectively employed SPE for GDGT degradation pathway studies by reducing oil contaminant interference [96], while Xiaoxue Wang et al. demonstrated SPE–MTBE equivalence in *Pyrococcus yayanosii* lipid diversity recovery through optimized protocols involving ethanol extraction and Ostro SPE phospholipid removal plate utilization [97].

Though archaeal lipid-specific SPE sorbent/eluent optimization remains unexplored, strategic adaptation of CC and HPLC solvent systems provides viable optimization pathways: normal-phase SPE cartridges may employ CC eluent references, while reverse-phase systems can utilize HPLC mobile phase analogs.

## 4. Structural Elucidation of Archaeal Lipids

The structural elucidation of archaeal lipids relies fundamentally on MS, though its efficacy is contingent upon the availability of reference compounds with unambiguously resolved chemical configurations. This identification workflow is inherently path-dependent, initiating with structurally characterized archetypes (e.g., archaeol, GDGTs, and hydroxylated derivatives), from which mass spectral signatures—including molecular ion mass-to-charge ratios, collision-induced dissociation (CID) fragmentation patterns, and isotopic fine structures—are systematically cataloged. These empirical datasets serve as critical benchmarks for subsequent analyses of unknown compounds within complex environmental matrices. To address the intrinsic limitations of MS in de novo structural resolution, nuclear magnetic resonance (NMR) spectroscopy remains indispensable. This section delineates the complementary roles of MS, NMR, and ancillary techniques in advancing the structural characterization of archaeal lipids, collectively driving progress in understanding their chemical structures.

### 4.1. Mass Spectrometry

MS coupled with chromatographic separations such as GC and HPLC has emerged as the cornerstone for identifying archaeal lipids, particularly GDGTs in environmental samples [98,99]. MS enables structural identification through the precise determination of molecular ion masses and analysis of diagnostic fragment ions, resolving unique features such as ether linkages, isoprenoid chain branching, and cyclization [100,101,102,103]. Ether bonds, which exhibit greater stability than bacterial/eukaryotic ester bonds, are cleaved under high-energy collisions (HCD), yielding glycerol backbone fragments and isoprenoid chain derivatives [99]. These isoprenoid moieties, characterized by methyl branches or cyclic structures, generate fragmentation patterns reflective of chain length, branching positions, and cyclization degree. In addition, MS^2^ and MS^3^ or multi-stage activation (MS^n^) mass analysis can reveal deeper and more unique fragment ion patterns [99]. Isotopic distributions of molecular ions further validate molecular formulas, particularly for GDGTs with complex methyl branching. Critically, archaeal lipids lack fatty acid-derived ions observed in bacterial or eukaryotic ester-bound lipids, providing a key discriminant in mixed lipid samples.

Advanced analytical platforms, including HPLC coupled with time-of-flight MS (HPLC–TOF–MS), high-temperature GC with flame ionization detector (HTGC–FID), and HPLC with atmospheric pressure chemical ionization MS (HPLC–APCI–MS), have significantly advanced GDGT research [99]. Among these, HPLC–APCI–MS with selected ion monitoring (SIM) has become indispensable in biogeochemical studies, enabling targeted quantification of known GDGTs [104]. Compared to the SIM mode, the multiple reaction monitoring (MRM) mode developed by Yufei Chen et al. exhibits higher sensitivity for detecting trace amounts of GDGTs in environmental samples [105]. Recent innovations, such as atmospheric pressure photoionization Fourier-transform ion cyclotron resonance MS (APPI-FTICR MS), enhance molecular characterization through toluene-mediated proton transfer, generating stable pseudo-molecular ion ([M + H]^+^) [99]. While FTICR MS offers unparalleled resolution, its operational costs limit widespread adoption. High-resolution Orbitrap MS (~250,000 resolution at *m*/*z* 400) provides a cost-effective alternative for marine sediment analyses, particularly when integrated with HPLC [106]. Simplified sample preparation and the LC–MS method optimization have further accelerated GDGT research. Recent methodological advances include the use of C46-GDGT internal standard [104], tandem hydrophilic interaction liquid chromatography (HILIC) columns for isomer separation [107], and APPI-FTICR MS for rapid biomarker screening without chromatographic separation [108]. Kai P. Law et al. developed a novel analytical protocol based on HPLC–ion mobility mass spectrometry to enhance the coverage of the lipidome and characterize the conformations of archaeal lipids by their collision cross-sections (CCSs), and they comprehensively characterized the lipidome of the SCM1 strain, uncovering a potentially novel lipid candidate through negative ionization analysis [109]. Reviews have discussed the identification and quantitative analysis of archaeal lipids using MS in detail [17,80,98,99]; therefore, this topic will not be further elaborated in this article.

While MS-based approaches dominate archaeal lipid analysis, persistent challenges hinder their full potential. First, the lack of comprehensive, archaeal-specific lipid databases impedes high-throughput lipidomics. Second, only a limited subset of archaeal lipids (e.g., GDGTs) have been structurally characterized. Additionally, due to the limited commercial availability of cyclic/branched GDGT isomer standards, the scarcity of reference compounds undermines quantitative accuracy. Addressing these gaps necessitates interdisciplinary efforts to expand spectral libraries, synthesize isomer standards, and optimize hybrid separation-MS workflows.

### 4.2. Nuclear Magnetic Resonance

NMR spectroscopy, distinguished by its non-destructive nature, high sensitivity, and multi-parameter analytical capabilities, remains indispensable for resolving the molecular structures of archaeal lipids. By preserving sample integrity while elucidating structural details through chemical shifts, coupling constants, and relaxation times, NMR provides critical insights into rare or low-abundance archaeal lipids.

Early structural studies on *H*. *cutirubrum* lipids in 1962 predated widespread NMR accessibility, relying instead on ^32^P-labeled hydrolysis products, chromatographic behavior, and infrared spectroscopy to exclude fatty acid ester linkages [23]. Subsequent elemental analysis of carbon, hydrogen, and phosphorus further supported the inference that the membrane lipids of *H. cutirubrum* adopt a glycerol–dialkyl diether structure [23]. Subsequent chemical degradation with BCl₃ and HI yielded alkyl iodides, analyzed via NMR to confirm dihydrophytanyl chains through methyl (*δ* = 0.836 ppm), methylene, and iodine-associated carbon signals. These findings established the glycerol–dialkyl diether framework of archaeal phospholipids, later confirmed via optical rotation [110].

By the 1970s, the widespread adoption of NMR enabled Mario de Rosa’s team to investigate the membrane lipids of the thermoacidophilic archaeon strain MT3 [24,111,112,113,114]. Their work culminated in the first NMR-confirmed identification of GDGT as the core lipid, with five- or six-membered rings embedded, significantly enhancing membrane thermal stability. This discovery expanded our understanding of archaeal lipid diversity and established a paradigm for analyzing complex lipid structures.

**Figure 6:**
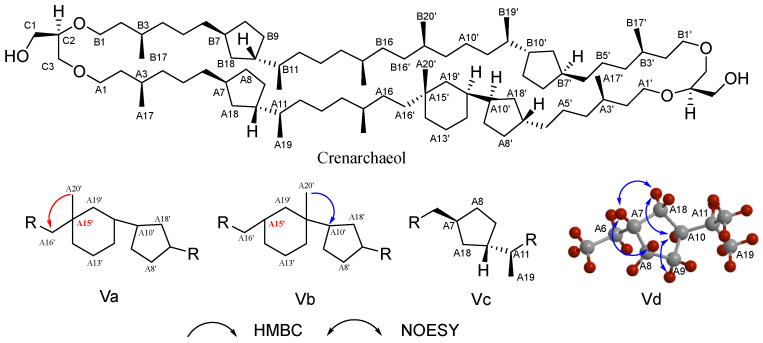
The structural elucidation of crenarchaeol, key HMBC (arrows pointing from H to C), and NOESY correlations; HMBC experiments confirmed the connectivity of its six-membered ring system (Va and Vb), and NOESY correlations established the stereochemical configuration of its cyclopentane moieties (Vc and Vd).

The structural complexity of archaeal lipids poses significant challenges for NMR analysis. The structure elucidation of crenarchaeol (a signature GDGT lipid in marine pelagic thermophilic archaea), first achieved by Jaap’s team with subsequent refinements, serves as our example for demonstrating NMR-based archaeal lipid analysis [115]. Basic chemical shift information is provided through ¹H NMR and ^13^C NMR spectra to identify methyl, methylene, methine, and quaternary carbon signals. In Heteronuclear Multiple Quantum Correlation (HMQC) spectrum, direct carbon–hydrogen connections can be correlated to confirm the ether bond connection sites of the glycerol unit and the diphytanyl chains. In ¹H NMR, a singlet methyl peak at *δ* = 0.836 ppm (A20’) and a doublet methyl peak at *δ* = 0.844 ppm are observed, whereas in the GDGT4 spectrum, these positions appear as an overlapping peak with an integral value of 12 hydrogen atoms. Coupled with a quaternary carbon signal at *δ* = 22.39 ppm in ^13^C NMR (A15’), these observations suggest the presence of a cyclohexane ring. For this cyclohexane ring formed after the cyclization of the biphytanyl chain, two possible configurations exist (Figure 6, Va and Vb). Heteronuclear Multiple Bond Correlation (HMBC) experiments effectively distinguished these possibilities: the singlet at 0.836 ppm (A20’) did not show correlation with the adjacent carbon atom (A10’) on the cyclopentane ring, which is inconsistent with the anticipated skeleton Vb; instead, it exhibited correlation with carbon atom A16’, aligning with the characteristics of configuration Va. The presence of a cyclohexane ring is thus confirmed (Figure 6). Further, the coupling relationships between protons are resolved, clarifying the connection order of protons within the cyclopentane and cyclohexane rings through Correlated Spectroscopy (COSY) and Total Correlation Spectroscopy (TOCSY). The stereochemistry of the cyclopentane ring in crenarchaeol was confirmed to be identical to that of GDGT-4 through Nuclear Overhauser Effect Spectroscopy (NOESY) analysis, which revealed characteristic Nuclear Overhauser Effect (NOE) correlations among the A7/8/9/10/18 protons (Figure 6). The configurations of A11(*S*) and A15(*R*) in the cyclohexane ring, established by analyzing the coupling constants of adjacent protons, are consistent with the proposed biosynthesis mechanism involving A15/A19 cyclization. By comparing the NMR data of GDGT-4 with NOESY signals, it is confirmed that the glycerol unit is *sn*-2,3-*O*-alkylated (*R* configuration).

GDNT (glycerol dialkyl nonitol tetraether), a tetraether lipid commonly found in the lipids of thermophilic and acidophilic archaea, is distinguished from GDGTs by its unique asymmetric headgroup (calditol) (Figure 2). The earliest structural elucidation of GDNT dates back to 1980, when Mario De Rosa and colleagues pioneered its characterization [116]. However, it was not until 2008 that Moira L. Bode and collaborators successfully obtained complete ^13^C and 2D-NMR data for GDNT isolated from *Sulfolobus metallicus*, enabling a detailed structural validation [117]. In the article delving into the structural elucidation of the head group of GDNT-β-Glu in *S*. *metallicus*, the connectivity between the β-glucose and calditol units within the molecule was established through meticulous HMBC and Rotating Frame Overhauser Effect Spectroscopy (ROESY) experiments, ultimately culminating in the confirmation of the compound’s chemical structure.

NMR advancements have unveiled novel lipid structures from diverse archaeal species: including glycocardiolipin variants in *Haloferax volcanii* [118], tetraether lipids featuring butanetriol and pentanetriol backbones in *Methanomassiliicoccus luminyensis* [119], novel head group-modified membrane lipids (bearing N,N-dimethylamino and N,N,N-trimethylaminopentyl moieties) identified in *Methanospirillum hungatei* [120], along with phosphothioglycolipids and di-*O*-alkyl glyceroglycolipid analogs discovered in *Halobacterium salinarum* [121]. These findings collectively underscore NMR’s pivotal role in expanding our understanding of archaeal lipid diversity and evolutionary adaptations.

However, the complex structures of archaeal lipids still pose significant challenges, especially with the identification of cyclic structures and regioisomers in the GDGTs. The introduction of five- or six-membered rings in the GDGTs leads to extensive overlap of hydrogen and carbon signals; moreover, the presence of regioisomers (such as differences in cyclohexane substitution sites in crenarchaeol) relies on high-resolution 2D NMR spectra for differentiation. A 2021 study revised crenarchaeol’s structure, highlighting cyclohexane substitution heterogeneity and underscoring the complexity of archaeal lipid analysis [122]. Beyond core lipids, NMR plays a vital role in polar headgroup characterization. In ^31^P NMR, the connection mode of the polar head phosphate group is quickly judged through characteristic phosphate signals and based on NOE, the stereoconfiguration of the polar head glycosyl group is determined. For example, in the identification of glycosyl moieties in the lipid structure of *Thermoplasma acidophilum*, the NOE signals observed in the ROESY spectrum not only confirmed the configuration of the glycosyl groups but also provided clues about the linkage between the glycosyl moieties and the lipid backbone [123]. Particularly, the NOE signal between H-1 and H-1’ indicated the presence of a glycosidic bond.

NMR spectroscopy further serves as a powerful tool for probing the stereochemical properties of archaeal lipids. Advances in chiral derivatization methodologies permit researchers to functionalize DGGGOH—a pivotal biosynthetic intermediate in lipid metabolism—and systematically analyze its characteristic ^1^H NMR patterns after Mosher’ method against authentic stereochemical standards [82,124]. This approach provides critical insights into the enantiomeric configuration and biosynthetic pathways of these evolutionarily conserved lipid frameworks. However, facing challenges such as cyclic structures, regioisomers, and stereochemistry of core lipid, multidimensional technology collaboration (such as Density Functional Theory (DFT) calculations and synthetic controls) is still needed to improve accuracy.

### 4.3. Spray Reagents

**Figure 7:**
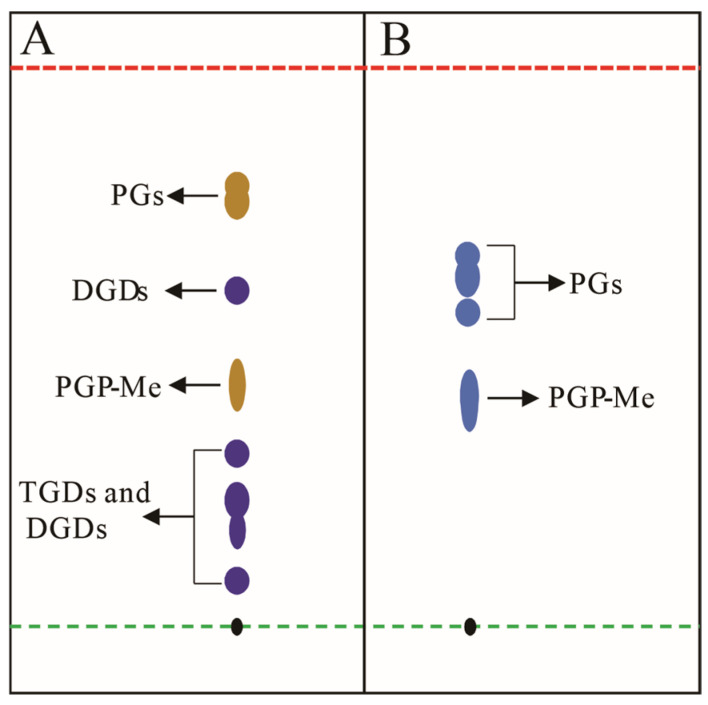
Schematic diagram illustrates conceptual TLC analysis of halophilic archaeal lipid extractions; (**A**) lipid extract from *Natronorubrum aibiense* stained with 0.5% α-naphthol reagent, showing dark yellow spots corresponding to phosphatidylglycerol derivatives (PGs), and purple spots representing triglycosyl diethers (TGDs) and diglycosyl diethers (DGDs) [128]; (**B**) lipid extract from *Haloterrigena longa* stained with Zinzadze reagent, where blue spots indicate phosphatidylglycerol species (PGs) [129]. Both analyses employed chloroform/methanol/acetic acid/water (85:22.5:10:4, *v*/*v*) as the developing solvent. Black spots denote the TLC origin. Green and red dashed lines demarcate the sample application origin and solvent front, respectively. Abbreviations: PGP-Me (phosphatidylglycerol phosphate methyl ester); PGs (phosphatidylglycerols); TGDs (triglycosyl diethers); DGDs (diglycosyl diethers). Note: *Rf* values shown do not represent actual experimental data.

TLC, a pivotal tool in archaeal lipid research (Section 2.3), necessitates specialized staining reagents for visualizing colorless or non-fluorescent lipids (e.g., phospholipids and glycolipids) [125,126,127]. Reagents are categorized by specificity (universal vs. targeted) and impact on sample integrity (destructive vs. non-destructive), with non-destructive methodologies preferred for preserving structural fidelity [85]. Universal reagents include iodine vapor, which forms reversible complexes with lipid double bonds, and destructive sulfuric acid–ethanol charring (120 °C), though the latter risks structural alteration. For archaeal lipids analysis, specific chemical reagents are particularly valuable for distinguishing broad lipid classes such as glycolipids and phospholipids (Figure 7). Chemical staining combined with TLC, especially 2D TLC has proven instrumental in archaeal chemotaxonomy. For example, in halophilic archaeal lipid identification, different staining agents can be used to detect different types of lipids [88]. Spraying the TLC plate with a phosphate-specific reagent (such as Dittmer–Lester reagent) until fully moistened reveals deep blue phospholipid spots against a white background [127]. The 0.5% α-naphthol reagent can be used to detect glycolipids by spraying, air-drying, and then spraying a small amount of concentrated sulfuric acid–ethanol (1:1, *v*/*v*) solution, followed by baking at 120 °C for 5–10 min until fully stained, at which point the glycolipid spots appear bluish-purple, while phospholipids turn yellow (Figure 7A) [83]. For the detection of total lipids, a general concentrated sulfuric acid–ethanol (1:1, *v*/*v*) staining agent can be used.

The choice of TLC reagents must balance specificity, sensitivity, and sample preservation, while integrating preliminary screening with downstream analyses (e.g., MS). Although there is currently no specific chromogenic agent for archaeal lipids, one can refer to some newly developed, broadly applicable specific chromogenic agents for experimentation, such as those specifically designed to detect phosphate groups. Future advancements, such as high-specificity fluorescent probes or nanomaterial-enhanced staining technologies, hold promise for overcoming traditional sensitivity and applicability limitations, offering robust support for archaeal lipidomic research.

### 4.4. Other Techniques

Prior to NMR’s dominance, early archaeal lipid structural characterization integrated infrared spectroscopy (IR), optical rotation (OR), chemical derivatization, and MS with chemical derivatization and fragment pattern comparisons to infer lipid structures, which has been discussed in Section 4.2. IR served as a cornerstone for preliminary identification, detecting key functional groups. OR, paired with synthetic standards, resolved chiral centers.

As early as 1992, David H. Thompson and colleagues combined Raman Spectroscopy, ^31^P NMR, X-ray scattering, and electron microscopy, demonstrating that archaeal membrane lipids span a monolayer membrane with their two polar headgroups located on opposite sides of the membrane interface [130]. In 2003, Parkson Lee-Gau Chong and colleagues provided detailed insights into the phase behavior, conformation, and structure of polar lipid fraction E (PLFE) under varying temperature and pressure conditions by combining information from SAXS (Small-Angle X-ray Scattering) and high-pressure FT-IR (Fourier Transform Infrared Spectroscopy) techniques [131].

## 5. Conclusions and Perspectives

Over the past decades, significant progress has been made in the chemical research of archaeal lipids, with numerous unique lipid structures identified across diverse archaeal taxa and environmental matrices. Yet, as highlighted in previous Sections, foundational research—such as the structural elucidation of archaeal lipids—remains limited.

This review establishes a methodological framework for archaeal lipid research based on the core technical concept of natural product chemistry—extraction, separation, and structural identification. The framework is systematically elaborated as follows: (1) extraction: the mechanistic analysis of existing methods guides optimized protocol selection, particularly for enhancing yields in pure culture studies; (2) separation: a tiered approach combines bulk fractionation (column chromatography/SPE) with precision purification (HPLC) for polarity-based lipid isolation; (3) structural identification: advances in NMR applications are discussed, particularly stereochemical configuration analysis through specialized techniques.

Despite the systematic review proposing several methodological applicability suggestions, the scientific and industrial applications of archaeal lipids still encounter challenges and bottlenecks that require urgent attention.

(1) Standardization deficits: the paucity of commercially available archaeal lipid reference standards critically undermines the reliability of quantitative analyses and chemotaxonomic applications, necessitating the urgent development of authenticated compound libraries.
(2) Methodological limitations: while the B&D method is widely adopted, studies on pure archaeal cultures reveal that pretreatments, such as high-pressure homogenization, ultrasonic disruption, or freeze–thaw cycles, significantly enhance lipid yields, underscoring the need for the systematic optimization of extraction protocols.
(3) Taxonomic and evolutionary ambiguities: the ongoing discovery of novel ether-linked lipids continues to expand our understanding of archaeal membrane diversity, simultaneously raising unresolved questions. These encompass not only evolutionary drivers for the unique structural divergence of archaeal lipids from bacterial/eukaryotic counterparts but also uncertainties regarding lipid-based taxonomic classifications. For instance, the proposed chemotaxonomic frameworks for halophilic archaea remain contentious due to interspecies variability in membrane lipid composition—a discrepancy yet to be resolved through comprehensive comparative studies.
(4) Analytical Complexity: compared to other natural product classes, archaeal lipid chemistry remains underexplored, a disparity exemplified by the structural revision of crenarchaeol. Initially characterized in 2002, its cyclohexane ring configuration required nearly two decades for correction via advanced NMR methodologies, reflecting both the technical intricacy of lipid analysis and an insufficient investigative focus.

The chemical exploration of archaeal lipids not only elucidates fundamental questions regarding life’s origins and extremophile adaptations but also furnishes novel molecular scaffolds for synthetic biology and biomaterial innovation. To catalyze transformative progress, this field necessitates concerted interdisciplinary efforts integrating chemists, microbiologists, and analytical specialists in MS/NMR technologies. Through such collaborative frameworks, research may evolve from descriptive structural analyses to functionally oriented investigations, ultimately unlocking the full biotechnological potential inherent to archaeal lipid systems.

## Data Availability

Not applicable.

## Abbreviations

The following abbreviations are used in this manuscript:

APCI	Atmospheric Pressure Chemical Ionization
APPI	Atmospheric Pressure Photoionization
ASE	Accelerated Solvent Extraction
B&D	Bligh–Dyer
CC	Column Chromatography
CCSs	Collision Cross-Sections
CID	Collision-induced Dissociation
CL GDGTs	Core lipids Glycerol Dialkyl Glycerol Tetraethers
COSY	Correlated Spectroscopy
CTAB	Cetyltrimethylammonium Bromide
DCM	Dichloromethane
DFT	Density Functional Theory
DGGGOH	sn-2,3-di-O-Geranylgeranylglyceryl Alcohol
DMSO	Dimethyl Sulfoxide
DGDs	Diglycosyl Diethers
ESI	Electrospray Ionization
FID	Flame Ionization Detector
FTICR	Fourier Transform Ion Cyclotron Resonance
FT-IR	Fourier Transform Infrared Spectroscopy
G1P	Glycerol-1-Phosphate
G3P	Glycerol-3-Phosphate
GC	Gas Chromatography
GDGTs	Glycerol Dialkyl Glycerol Tetraethers
GDNT	Glycerol Dialkyl Nonitol Tetraether
GFF	Glass Fiber Filters
HPH	Hexose-phosphohexose headgroups
HCD	High Energy Collision Dissociation
HILIC	Hydrophilic Interaction Liquid Chromatography
HMBC	Heteronuclear Multiple Bond Correlation
HMQC	Heteronuclear Multiple Quantum Correlation
HPLC	High-Performance Liquid Chromatography
HPTLC	High-Performance Thin-Layer Chromatography
HTGC	High-Temperature Gas Chromatography
I-GDGTs	Isoprenoid Glycerol Dialkyl Glycerol Tetraethers
IPLs	Intact Polar Lipids
IR	Infrared Spectroscopy
LC	Liquid Chromatography
LLE	Liquid–Liquid Extraction
LVI	Large-Volume Injection
MALDI-MS	Matrix-Assisted Laser Desorption/Ionization Mass Spectrometry
MeOH	Methanol
MRM	Multiple Reaction Monitoring
MS	Mass Spectrometry
MTBE	Methyl Tertiary-Butyl Ether
NMR	Nuclear Magnetic Resonance
NOE	Nuclear Overhauser Effect
NOESY	Nuclear Overhauser Effect Spectroscopy
NP	Normal-phase
NS	Non-Significant
OR	Optical Rotation
PLFE	Polar lipid fraction E
PGs	Phosphatidylglycerols
PGP-Me	Phosphatidylglycerol Phosphate Methyl Ester
ROESY	Rotating Frame Overhauser Effect Spectroscopy
RP	Reverse-phase
RSD	Relative Standard Deviation
SAXS	Small-Angle X-ray Scattering
SIM	Selected Ion Monitoring
SPE	Solid-Phase Extraction
2D TLC	Two-Dimensional Thin-Layer Chromatography
TCA	Trichloroacetic Acid
TEX86	TetraEther IndeX of 86 Carbons
TLC	Thin-Layer Chromatography
TOCSY	Total Correlation Spectroscopy
TOF	Time-of-Flight
TGDs	Triglycosyl Diethers
UAE	Ultrasonic-Assisted Extraction
UK	Unsaturated Ketone Index
UV	Ultraviolet
